# Association of Lung Immune Prognostic Index (LIPI) with Disease Control Rate and Progression-Free Survival in Patients with Soft-Tissue Sarcoma Treated with Immunotherapy in Early-Phase Trials

**DOI:** 10.3390/cancers16234053

**Published:** 2024-12-03

**Authors:** Marianne Zoghbi, Brina A. Patel, Matthieu Roulleaux Dugage, Laura Mezquita, Rastilav Bahleda, Armelle Dufresne, Mehdi Brahmi, Isabelle Ray-Coquard, Patricia Pautier, Jean-Yves Blay, Axel Le Cesne, Christophe Massard, Benjamin Besse, Edouard Auclin, Elise F. Nassif Haddad

**Affiliations:** 1 Department of Sarcoma Medical Oncology, University of Texas MD Anderson Cancer Center, Houston, TX 77030, USA; 2 Département d’Innovation Thérapeutique et d’Essais Précoces, Gustave Roussy, 94805 Villejuif, France; 3 Department of Medical Oncology, IDIBAPS, Hospital Clínic, 08036 Barcelona, Spain; 4 Department of Medicine, University of Barcelona, 08036 Barcelona, Spain; 5 Département d’Oncologie Médicale, Centre Léon Bérard, 69008 Lyon, France; 6 Département de Médecine Oncologique, Gustave Roussy, 94805 Villejuif, France; 7 International Department, Gustave Roussy, 94805 Villejuif, France; 8 Department of Medical Oncology, Institut Bergonié, 33076 Bordeaux, France; ed.auclin@gmail.com; 9 Department of Investigational Cancer Therapeutics, University of Texas MD Anderson Cancer Center, Houston, TX 77030, USA

**Keywords:** LIPI, immunotherapy, soft-tissue sarcoma, clinical trials, biomarker, prognostic, predictive

## Abstract

Background: The efficacy of immunotherapies in soft-tissue sarcomas (STSs) is limited, and biomarkers of response are lacking. The lung immune prognostic index (LIPI) is a prognostic biomarker used with immunotherapy across cancer types. This study investigates the association of LIPI with the disease control rate (DCR) and progression-free survival (PFS) in patients with STS treated with immunotherapy versus other therapies in early-phase trials. Methods: This post hoc analysis was conducted with patients with STS from Gustave Roussy and Centre Léon Bérard between January 2012 and June 2021. The LIPI was calculated based on a derived neutrophil-to-lymphocyte ratio > 3 and elevated lactate dehydrogenase. Patients were categorized based on treatment (immunotherapy or other) and LIPI (good, intermediate, or poor). DCR was defined as the sum of stable disease and complete and partial response. Results: A total of 82 patients were enrolled in immunotherapy trials and 126 in the other therapy trials. In the immunotherapy group, DCR was higher in patients with good LIPI (76%; *n* = 23/30) compared with the intermediate (50%; *n* = 13/26) and poor LIPI groups (8%; *n* = 1/12; *p* < 0.001). The other-therapy group did not show significant differences in DCR by LIPI: DCR was 70% (*n* = 48/69), 70% (*n* = 21/30), and 60% (*n* = 6/10) in patients with good, intermediate, and poor LIPI, respectively (*p* = 0.86). In multivariate analyses, LIPI was independently associated with PFS in the immunotherapy group (hazard ratio = 5.97, *p* = 0.0001) and not in the control group (*p* = 0.71). Conclusions: LIPI is a significant independent prognostic marker for DCR in patients with STS treated with immunotherapy. In early-phase trials, LIPI could be used as a screening tool for stratification at inclusion. High neutrophil levels, which correlate with a poorer LIPI score, are likely associated with immunotherapy resistance. This relationship could explain the statistical impact of poor LIPI in the immunotherapy group.

## Simple Summary

The study aims to evaluate the prognostic and predictive significance of the lung immune prognostic index (LIPI) in patients with soft-tissue sarcomas (STSs) undergoing immunotherapy compared with other treatments in early-phase clinical trials. LIPI, which has been a proven prognostic tool in various other cancer types, may also serve as a valuable biomarker for guiding treatment decisions in STS. Our study showed that among patients with STS treated with immunotherapy, LIPI appeared to be a promising predictive marker of disease control rate and a promising prognostic marker of progression-free survival and overall survival. Thus, LIPI could be used as a screening tool for patients with STS when considering an immunotherapy early-phase clinical trial. This is particularly significant given the limited response rates to current treatments for STS, highlighting the need for reliable prognostic tools.

## 1. Introduction

Soft-tissue sarcomas (STSs) are a challenging subset of cancer due to their rarity and heterogeneity. Currently, treatment for metastatic or advanced STS involves a combination of cytotoxic chemotherapies and broad multi-kinase inhibitors. Traditionally, the standard first-line systemic treatment for sarcomas has been anthracycline-based chemotherapy (doxorubicin), with response rates of only 12–24% [1,2,3]. Immunotherapy has emerged as a promising alternative; however, its effectiveness is currently under investigation, with mixed results from clinical trials and response rates ranging from 5% to 18% [4,5,6,7,8]. These results underscore the need for reliable prognostic tools to optimize patient outcomes.

One such tool is the lung immune prognostic index (LIPI), which has been shown to be an effective prognostic biomarker of the response to immunotherapy in other cancer types. The LIPI score is calculated based on specific blood biomarkers: the derived neutrophil-to-lymphocyte ratio (dNLR) and lactate dehydrogenase (LDH) levels. By investigating these biomarkers, the LIPI score provides insight into the systemic immune system, which is a key factor influencing tumor progression and patient outcomes [9,10]. Several studies have shown that LIPI is associated with outcomes in patients with immunotherapy for advanced non-small cell lung cancer (NSCLC), small cell lung cancer (SCLC), mismatch repair deficient tumors, urinary tract carcinoma, and even localized non-small cell lung cancer [9]. The predictive and prognostic impact of LIPI for patients with STS treated with immunotherapy has not yet been studied.

Our main objective was to identify the predictive significance of the LIPI score in patients with STS being treated with immunotherapy in comparison with other treatments as part of early-phase clinical trials. This retrospective study of patients with STS treated as part of early-phase trials aims to determine whether the LIPI score can serve as a reliable tool to guide treatment decisions.

## 2. Materials and Methods

### 2.1. Study Design

This study is a post hoc analysis with data gathered prospectively from clinical trials. The study expands upon a previously published cohort of patients included in early-phase trials at the Drug Development Department of Gustave Roussy and the early-phase trial unit of Centre Léon Bérard between 1 January 2012 and 31 December 2020. For this specific analysis, data collection was expanded to June 2021 for the inclusion of additional patients. Patients with gastrointestinal stromal tumors (GISTs), bone sarcomas, small round cell tumors, and those being treated in early-phase trials exploring purely diagnostic radiotracers were excluded to focus solely on patients with STS in therapeutic-intent trials.

Patients were classified into two groups depending on treatment modality: immunotherapy and other (control group). The immunotherapy trials were either immune checkpoint inhibitors (ICIs), alone or in combination, or modified T-cell receptor cellular therapies; the control group included targeted therapies, multi-kinase inhibitors, and cytotoxic chemotherapies. If patients received a combination of immunotherapy and another agent, they were classified within the immunotherapy group.

### 2.2. Data Collected

Data collected from the trials included histology, baseline clinical and biological characteristics at the start of the trial (age, sex, stage at inclusion, ECOG performance status (PS), number of prior systemic lines, prior treatment with anthracyclines, number of metastatic sites, and presence of liver and lung metastases), the Royal Marsden Hospital (RMH) score, and the dNLR [11]. The RMH score, a prognostic tool for early-phase trials, is calculated based on elevated LDH levels, albumin levels below 35 g/L, and the number of metastatic sites (greater than 2) [11]. RMH scores range from 0 to 3, with 0 being most favorable and 3 being least favorable, and patients with a lower RMH score tend to have longer survival in early-phase trials. All trial-related details were recorded following the specific protocol requirements, including the use of Common Terminology Criteria for Adverse Events (CTCAE) version 4 or 5 and the documentation of objective response rate (ORR) and disease control rate (DCR) according to either the Response Evaluation Criteria in Solid Tumors (RECIST) 1.1 or immune-related RECIST, as per protocol.

### 2.3. LIPI Score

Patients were also classified into subgroups dependent on their LIPI score (good, intermediate, poor). LIPI score is calculated based on dNLR and serum LDH level [9]. Good scores were defined as those with dNLR < 3 and LDH < Normal, intermediate scores were dNLR >3 or LDH > Normal, and poor scores were dNLR > 3 and LDH > Normal (Table S1). Patients were all included in clinical trials, and thus required a washout period before treatment initiation, and the measurement of dNLR was made after this washout period, at baseline, so the impact of other supportive medications such as Granulocyte Colony-Stimulating Factor (GCSF) on the measurement of dNLR in this cohort is anticipated to be minimal.

### 2.4. Statistical Analysis

For the description of patients’ characteristics, median (Interquartile Range, IQR) and proportions were used for continuous or categorical variables, respectively. Comparisons between subgroups were made with the Wilcoxon–Mann–Whitney test for continuous variables and the chi2-test (or Fisher’s exact test, if appropriate) for categorical variables. The primary endpoint was DCR according to RECIST 1.1. DCR was defined as the sum of complete response (CR), partial response (PR), and stable disease (SD) according to RECIST 1.1, with any SD duration as per each separate protocol. ORR was defined as the sum of CR and PR according to RECIST 1.1. A secondary analysis was performed with only SD that lasted at least 8 weeks.

Secondary endpoints included progression-free survival (PFS) and overall survival (OS). PFS was defined as the period from the start of ICI treatment to either disease progression (PD) or death. OS was defined as the duration from the initiation of ICI therapy to death from any cause. Kaplan–Meier estimates were used to calculate OS and PFS, with group comparisons conducted via the log-rank test. Follow-up duration was determined using the reverse Kaplan–Meier method.

We used univariate and multivariate Cox proportional hazards models to evaluate the associations between potential prognostic factors and survival outcomes. All statistical analyses were conducted with RStudio software, with a significance threshold set at *p* < 0.05 for all two-sided tests. The multivariable Cox models presented in the Supplement files are constructed with the OS endpoint, and variables with *p*-value < 0.05 in univariate analysis or considered to be clinically meaningful were included in the model.

### 2.5. Ethical Consideration

At the time of their individual trial enrollment, each patient provided their informed consent for participation in trials. The use of the previously obtained data was reported to the French National Data Registry in compliance with MR-004 guidelines, and consent was waived for this retrospective investigation.

## 3. Results

### 3.1. Patient Cohorts

Our final population included 208 patients; 82 patients received immunotherapy, and 126 received other treatment types (Figure S1: Flowchart). The median age in the whole cohort was 55 years old, with more female (*n* = 111) than male (*n* = 97) patients. In the immunotherapy cohort, the median age was 55 years old, with fewer female (*n* = 38) than male (*n* = 44) patients. In the control cohort, the median age was 58 years old and there were more female (*n* = 73) than male patients (*n* = 53; Table 1 and Table S2).

In the immunotherapy cohort, the most frequently represented types of STS were other histotypes (*n* = 28), leiomyosarcoma (*n* = 22), well- and de-differentiated liposarcoma (WD/DDLPS; *n* = 9), synovial sarcoma (*n* = 7), angiosarcoma (*n* = 6), undifferentiated pleomorphic sarcoma (UPS; *n* = 5), and myxoid liposarcoma (*n* = 5). In the control cohort, most patients were treated for WD/DDLPS (*n* = 45), leiomyosarcoma (*n* = 32), other histotypes (*n* = 21), UPS (*n* = 11), myxoid liposarcoma (*n* = 6), angiosarcoma (*n* = 6), and synovial sarcoma (*n* = 5; Figures S1 and S2).

The stage at inclusion in the whole cohort was locally advanced and metastatic for 27 (13%) and 181 (87%) patients, respectively. Specifically, patients’ tumors were locally advanced and metastatic in 10 (12%) and 72 (88%) cases, respectively, for the immunotherapy cohort and 17 (13.5%) and 109 (86.5%), respectively, for the control cohort. There was a median of 2.5 prior systemic lines in the whole cohort: 3 prior lines in the immunotherapy cohort and 2 in the control cohort. In the whole cohort, 168 patients received prior anthracycline: 74 patients (90%) in the immunotherapy cohort and 94 patients (75%) in the control cohort. In the immunotherapy cohort, 71 patients received combination treatments with at least two drugs (87%); among them, 54 patients received a combination of immunotherapy and another agent (66%). Only 38 patients received combination treatment in the control cohort (30%).

### 3.2. Distribution of LIPI

Due to missing data (either LDH, lymphocyte, or neutrophil values), LIPI was evaluable for only 119 patients in the control group and 69 patients in the immunotherapy group. In the entire cohort, 103 (55%), 59 (31%), and 26 (14%) patients had a good, intermediate, and poor LIPI, respectively. The LIPI distribution varied between the immunotherapy and the control cohort, with the immunotherapy group showing a significantly lower proportion of patients with good LIPI and a higher proportion of patients with intermediate LIPI compared with the control group (*p* < 0.001). In the immunotherapy cohort, 30 (43%), 26 (38%), and 13 (19%) patients had a good, intermediate, and poor LIPI, respectively. In the control cohort, 73 (61%), 33 (28%), and 13 (11%) patients had a good, intermediate, and poor LIPI, respectively.

The baseline characteristics according to LIPI groups in each cohort are summarized in Table 1. ECOG PS > 0 was significantly associated with LIPI distribution in both cohorts. In the immunotherapy cohort, among patients with evaluable LIPI, 45 patients (65%) had a PS > 0, with more patients with a good LIPI (*n* = 17) compared with the intermediate (*n* = 15) and poor LIPI groups (*n* = 13; *p* = 0.007). In the control cohort, among patients with evaluable LIPI, 70 patients (59%) had a PS > 0, with a higher number in the good LIPI group (*n* = 33) compared with the intermediate (*n* = 26) and poor LIPI groups (*n* = 11; *p* = 0.001).

In the immunotherapy cohort, patients with good LIPI scores were more likely to have albumin levels greater than 35 g/L (*p* = 0.047). Also, histological analysis indicated a significant association between histology type and distribution of LIPI in this cohort (*p* = 0.022).

In the control cohort, female sex was significantly associated with poorer LIPI (*p* = 0.03). Additionally, LIPI was significantly associated with genomic profile (*p* = 0.01), whereby patients with translation-related sarcomas had a better LIPI than those with complex genomic sarcomas: among patients with a complex karyotype, 29 (48%), 21 (35%), and 10 (17%) had a good, intermediate, and poor LIPI, respectively, compared with 44 (75%), 12 (20%), and 3 (5%) patients in the translocation-related sarcoma group with good, intermediate, and poor LIPI, respectively. However, in the immunotherapy cohort, the trend was reversed, with patients having a complex karyotype tending to have a better LIPI compared with those with translation-related sarcoma.

### 3.3. Response Rates

**Figure 1:**
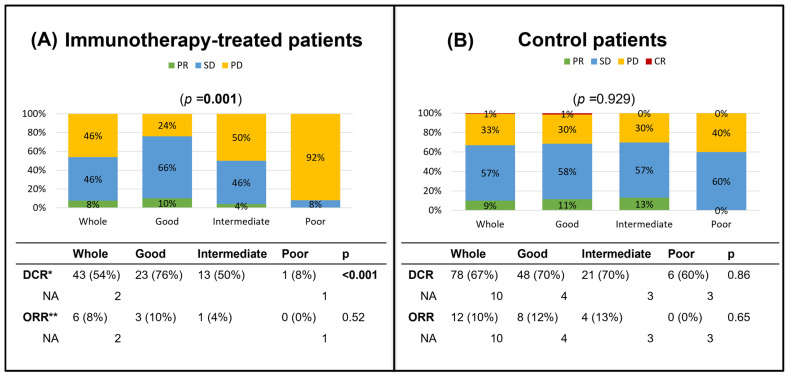
Barplot of responses by LIPI group from the immunotherapy and control cohorts. (**A**) Best response by LIPI score in the immunotherapy cohort. (**B**) Best response by LIPI score in the control cohort. * Disease control rate = stable disease + partial response + complete response. ** Objective response rate = partial response + complete response. Abbreviations: CR, complete response; DCR, disease control rate; ORR, objective response rate; PD, progressive disease; PR, partial response; SD, stable disease.

Among patients with evaluable response in the immunotherapy cohort, the DCR was notably higher in patients with good LIPI (76%; *n* = 23/30) compared with intermediate (50%; *n* = 13/26) and poor LIPI groups (8%; *n* = 1/12; *p* < 0.001). However, ORR was not associated with a significant difference among LIPI groups: 10% (*n* = 3/30), 4% (*n* = 1/26), and 0% (*n* = 0/12) in the good, intermediate, and poor LIPI groups, respectively (*p* = 0.52). Specifically, in patients with good LIPI, 10% (*n* = 3/30) had PR, 66% (*n* = 20/30) had SD, and 24% (*n* = 7/30) had PD as their best radiographic responses. In patients with intermediate LIPI, 4% (*n* = 1/26) had PR, 46% (*n* = 12/26) had SD, and 50% (*n* = 13/26) had PD; whereas in patients with poor LIPI, none had a PR, 8% (*n* = 1/12) had SD, and 92% (*n* = 11/12) had PD (one non-evaluable) (Figure 1).

Conversely, the control cohort did not show any significant differences in DCR or ORR by LIPI. Among patients with an evaluable response, DCR was 70% (*n* = 48/69), 70% (*n* = 21/30), and 60% (*n* = 6/10) in the good, intermediate, and poor LIPI groups, respectively (*p* = 0.86). ORR was 12% (*n* = 8/69), 13% (*n* = 4/30), and 0% (*n* = 0/10) in good, intermediate, and poor LIPI groups, respectively (*p* = 0.65) In patients with good LIPI, 1% (*n* = 1/69) had CR, 11% (*n* = 7/69) had PR, 58% (*n* = 40/69) had SD, and 30% (*n* = 21/69) had PD (4 not evaluable). In patients with intermediate LIPI, 13% (*n* = 4/30) had PR, 57% (*n* = 17/30) had SD, and 30% (*n* = 9/30) had PD (3 not evaluable); whereas in patients with poor LIPI, none had a PR, 60% (*n* = 6/10) had SD, and 40% (*n* = 4/10) had PD (3 not evaluable) (Figure 1).

Taking into account patients with SD more than 8 weeks in the immunotherapy group, DCR was 73% (*n* = 22/30), 42% (*n* = 11/26), and 0% (*n* = 0/13) in the good, intermediate, and poor LIPI groups, respectively (*p* < 0.001). Concerning the control cohort, also among patients with SD more than 8 weeks, DCR was 66% (*n* = 48/73), 64% (*n* = 21/33), and 46% (*n* = 6/13) in the good, intermediate, and poor LIPI groups, respectively (*p* = 0.93; Table S3).

### 3.4. Progression-Free Survival

**Figure 2:**
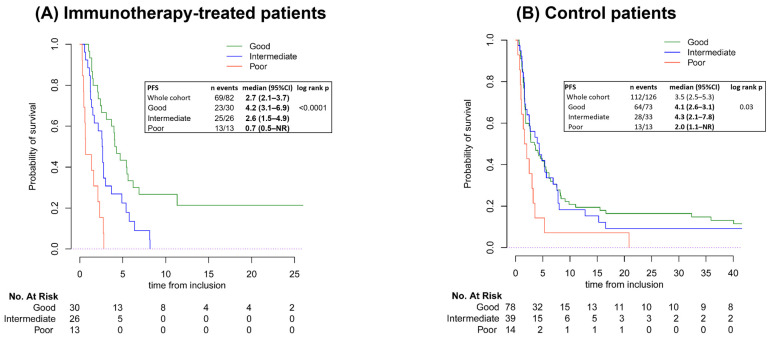
Kaplan–Meier curves of PFS by LIPI group. Both graphs are mapped according to the probability of survival and time from inclusion to denote number at risk. (**A**) PFS for the immunotherapy cohort, (**B**) PFS for the control cohort. Abbreviations: 95%CI, 95% Confidence Interval; NR, not reached; PFS, progression-free survival.

The median PFS was 2.7 months (95% Confidence Interval [95%CI]: 2.1–3.7) in the immunotherapy cohort and 3.5 months (95%CI: 2.5–5.3) in the control cohort. In the immunotherapy-treated patients, based on LIPI, the median PFS was 4.2 months (95%CI: 3.1–6.9), 2.6 months (95%CI: 1.5–4.9), and 0.7 months (95%CI: 0.5 to not reached [NR]) for patients with a good, intermediate, and poor LIPI, respectively (log-rank *p* < 0.001). In the control cohort, the median PFS was 4.1 months (95%CI: 2.6–3.1), 4.3 months (95%CI: 2.1–7.8), and 2.0 months (95%CI: 1.1–NR) for patients with a good, intermediate, and poor LIPI, respectively (Figure 2).

Univariate analysis for PFS in the immunotherapy and control group is given in Supplementary Table S4.

In multivariate analysis, factors significantly associated with shorter PFS in the immunotherapy cohort were LIPI intermediate (HR = 2.21; *p* = 0.01) and LIPI poor (HR = 3.89; *p* = 0.01) (Table 2). Conversely, in the control cohort, factors associated with improved PFS were a translocation-related genomic profile (HR = 0.59; *p* = 0.032) and combination treatments (HR = 0.59; *p* = 0.032; Table 2). LIPI was not associated with PFS in multivariate analysis in the control cohort (*p* = 0.7, Table 2).

Median PFS for patients who had SD more than 8 weeks in the immunotherapy cohort was 4.9 months (95%CI: 4.0–6.2), with a median of 5 (95%CI: 4.0–NR), 4.3 (95%CI: 2.7–NR), and 1.6 (95%CI: NR–NR) months in the LIPI good, intermediate, and poor groups, respectively. In the control group, median PFS for patients with SD more than 8 weeks was 5.6 months (95%CI: 4.4–7.6), with a median of 5.6 (95%CI:4.1–8.2), 5.4 (95%CI: 3.5–NR), and 3.4 (95%CI: 3.0–NR) months in the LIPI good, intermediate, and poor groups, respectively (Table S5).

In the analysis of PFS by dNLR, there were significant insights made in both cohorts. In the immunotherapy cohort, patients with a high dNLR had a longer median PFS (4.7 months) compared with those with low dNLR (4 months; log-rank *p* < 0.0001). In the control cohort, patients with a low dNLR did not have a significantly longer median PFS (4 months) than those with a high dNLR (2.6 months; *p* = 0.3; Figure S3).

### 3.5. Overall Survival

**Figure 3:**
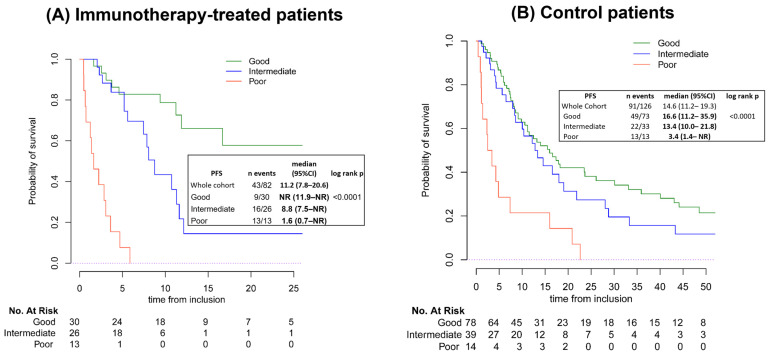
Kaplan–Meier curves of overall survival (OS) by LIPI group in the immunotherapy (**A**) and control (**B**) cohorts, respectively. Graphs are mapped according to the probability of survival and time from inclusion to denote number at risk. Abbreviations: 95%CI, 95% Confidence Interval; NR, not reached; PFS, progression-free survival.

Looking at OS by LIPI, the median OS was 11.2 months (95%CI: 7.8–20.6) in the immunotherapy cohort and 14.6 months (95%CI: 11.2–19.3) in the control cohort. In the immunotherapy cohort, OS was associated with LIPI: the median OS was not reached (95%CI: 11.9–NR), 8.8 months (95%CI: 7.5–NR), and 1.6 months (95%CI: 0.7–NR) in patients with good, intermediate, and poor LIPI, respectively (log-rank *p* < 0.0001). The median OS for patients in the control cohort was also associated with LIPI; patients with a good, intermediate, and poor LIPI had a median OS of 16.6 months (95%CI: 11.2–35.9), 13.4 months (95%CI: 10.1–21.8), and 3.4 months (95%CI: 3.4–NR, log-rank *p* < 0.0001), respectively (Figure 3).

Univariate analyses for OS in the immunotherapy and control groups are given in Supplementary Table S6 and Supplementary Table S7, respectively.

In multivariate analysis, regarding the immunotherapy cohort, factors significantly associated with shorter OS were synovial sarcoma histology (HR = 3.78, 95%CI:1.29–11.08, *p* = 0.015), intermediate LIPI (HR = 2.93, 95%CI: 1.15–7.49, *p*< 0.0001), and poor LIPI (HR = 15.67, 95%CI: 4.89–50.21, *p* < 0.0001). Albumin > 35 g/L was associated with a better OS (HR = 0.23, 95%CI: 0.1–0.54, *p* = 0.001) (Table S8). In the control cohort, factors significantly associated with shorter OS were synovial sarcoma histology (HR = 3.12, 95%CI: 11.07–9.09, *p* = 0.038) and performance status of 1 or more (HR = 1.92, 95%CI: 1.15–3.22, *p* = 0.013). Albumin > 35 g/L was also associated with a better OS (HR = 0.44, 95%CI: 0.25–0.75, *p* = 0.002) (Table S9).

Median OS for patients who had SD more than 8 weeks in the immunotherapy cohort was 16.7 months (95%CI: 11.2–NR), with a median not reached (95%CI:16.7–NR), 10.7 (95%CI: 5.6–NR), and 3.6 (95%CI: NR–NR) months in the LIPI good, intermediate, and poor groups, respectively. In the control group, median OS for patients with SD more than 8 weeks was 21.8 months (95%CI: 16–31.9), with a median of 30.1 (95%CI: 17.3–61), 14.5 (95%CI: 10.3–NR), and 10.4 (95%CI: 4.3–NR) months in the LIPI good, intermediate, and poor groups, respectively (Table S5).

Looking at OS by dNLR, in the immunotherapy cohort, patients with a low dNLR had a longer median OS (16.7 months) compared with those with high dNLR (4 months; log-rank *p* < 0.0001). In the control cohort, patients with a low dNLR did not have a significantly longer median OS (16.0 months) than those with a high dNLR (8.5 months; *p* = 0.09; Figure S4).

## 4. Discussion

Overall, the results of this study demonstrate that LIPI is significantly associated with DCR, PFS, and OS in patients with STS treated with immunotherapy early-phase trials and that it is an independent factor associated with PFS and OS in multivariate analyses in patients treated in immunotherapy trials for STS. In the immunotherapy cohort, the patients with good LIPI scores demonstrated better clinical outcomes. In patients with STS treated in early-phase trials with other types of treatment, LIPI was not associated with DCR or PFS and OS on multivariate analysis. Therefore, LIPI could be used as an easy tool to select patients for inclusion in early-phase trials with immunotherapy versus other types of treatments. Importantly, as the majority of patients with STS and poor LIPI had PD as the best response, it could raise the question of the relevance of including these patients in immunotherapy trials. Alternatively, LIPI could be considered as a stratification factor for immunotherapy-based clinical trials in patients with STS.

Immunotherapy for patients with STS has been generally disappointing, with low response rates across various trials. While the first trial assessing pembrolizumab in advanced STS (SARC028) demonstrated an ORR of 18% (*n* = 7/40) [12], the results highlighted that certain histotypes such as UPS and DDLPS may be more sensitive to immunotherapy with initial ORRs of 40% (*n* = 4/10) and 20% (*n* = 2/10), while other histologies were immune-resistant with ICIs (leiomyosarcomas and synovial sarcomas). Additionally, in a study evaluating durvalumab plus tremelimumab in advanced or metastatic STS, the ORR was 12%, and once again histology dependent. Specifically, patients with alveolar soft-part sarcoma exhibited the highest PFS at 12 weeks (80%) and had a notable ORR of 40%, while patients with liposarcomas had the lowest PFS at 12 weeks (17%) [13,14]. In another phase two trial examining the effect of nivolumab with or without ipilimumab treatment for metastatic sarcoma, the combination of nivolumab and ipilimumab displayed a higher ORR (16%) compared with nivolumab alone (5%). Notably, there was increased efficacy in UPS (23% ORR) and myxofibrosarcoma (29% ORR), suggesting heightened activity in certain sarcoma subtypes [15]. This aligns with results from previous studies, where responses to immunotherapy were histologically dependent. While UPS and alveolar soft-part sarcoma showed sensitivity to ICIs, other histological types such as leiomyosarcoma remained resistant. Overall, due to the broader challenge in immunotherapy for STS, there is a need for better biomarkers of response to identify which patients are more likely to benefit from immunotherapy, allowing for precise selection and potentially steering others toward clinical trials with other types of therapies.

In a series of retrospective studies, LIPI consistently demonstrated its utility as a significant screening tool for immunotherapy across various cancer types and treatments. In a study investigating the prognostic value of LIPI in patients with SCLC, results showed that patients with a good LIPI had a significantly longer median OS compared with those with an intermediate or poor LIPI, and this finding held true even in cases of extensive disease, where both PFS and OS were better in the good LIPI group [9]. Similarly, a study examining the association of LIPI with ICI outcomes in patients with advanced NSCLC demonstrated that a poor LIPI was associated with poorer outcomes: median OS did vary significantly across LIPIs (3, 10, and 34 months for poor, intermediate, and good, respectively), and median PFS differed across LIPIs (2.0, 3.7, and 6.3 months for poor, intermediate, and good, respectively). The results overall suggest a potential biological rationale for why patients with a high dNLR and LDH levels have poorer outcomes with ICIs, since a high dNLR could suggest a high systemic inflammatory state, and similarly, an increased LDH may reflect a higher tumor burden, both of which could contribute to ICI resistance [16]. Following this pattern, in a study examining the prediction of immunotherapy outcomes in older patients with solid tumors using LIPI, LIPI was significantly associated with OS: median OS was 20.7, 11.2, and 4.7 months in the good, intermediate and poor LIPI groups, respectively (*p* = 0.0003) [17]. In mismatch repair deficient tumors, a retrospective study found a strong association between LIPI and OS: the median OS was not reached in the good and intermediate groups but was only 3.3 months in the poor LIPI group. One year OS rates were 81%, 67.1%, and 21.4% for the good, intermediate, and poor LIPI groups, respectively. The median PFS was significantly shorter in the poor LIPI group (2.3 months) compared with 20.9 and 9.9 months for the good and intermediate groups, respectively [18]. In the localized setting, in a prospective trial examining the association between LIPI and durvalumab consolidation outcomes in patients with locally advanced NSCLC, baseline LIPI was associated with ICI benefit in NSCLC: the median OS (with a median follow-up of 19 months) was not attained, 47.0 months, and 18.1 months in the good, intermediate, and poor LIPI groups, respectively (*p* = 0.03). The control cohort did not experience any differences in survival or responsiveness [19]. The utility of the LIPI score was also proven in a study examining the prognostic value of the LIPI for patients treated with ICIs for advanced or metastatic urinary tract carcinoma. Patients treated with ICIs and a good LIPI had better clinical OS and PFS than those with a poor LIPI. Furthermore, data from patients with advanced NSCLC from three prospective trials (IMpower150, IMpower131, IMpower130) validated the prognostic role of LIPI in clinical trial datasets [20]. Our work adds a new histology to these studies, and these findings are consistent across the different treatment cohorts tested, underscoring LIPI’s ability as a screening tool for response to immunotherapy across cancer types. While LIPI appears to be prognostic only in NSCLC, it may be predictive in patients with STS.

In our control cohort, there was an association between LIPI poor and OS and PFS in univariate analysis, which was lost in multivariate analyses. This likely reflects some interaction between LIPI groups in this cohort and other factors. The immunotherapy and control cohorts were different since the immunotherapy cohort had slightly more prior lines of treatment, which did include a higher proportion of anthracycline-treated patients. Moreover, the control cohort had more patients with good LIPIs, which consisted of 60% of the cohort, whereas it was more balanced in the immunotherapy cohort. We do note that even in univariate analyses, LIPI did not stratify patients well between LIPI intermediate and good in our cohort for either OS or PFS, and the significant difference seen in univariate analysis reflects the poor outcomes of the LIPI poor group, which constituted only 10% of this cohort. However, LIPI itself may be a predictive variable for patients with STS regardless of the treatment methods, and we may lack power in our current control group to show this.

Recent studies on the tumor microenvironment have revealed new insights into antitumor activity and biomarker selection, specifically focusing on the role of tertiary lymphoid structures (TLSs), which are organized immune cell aggregates at tumor sites [21,22,23,24,25]. Clinical application of these findings can enhance patient selection for immunotherapy by utilizing TLS identification, which can be performed in standard pathology laboratories. A recent study on patients with advanced STS demonstrated that selecting those with TLSs for anti-programmed cell death protein 1 therapy significantly improved response rates and PFS [26]. This underscores the potential of TLS testing in identifying patients with sarcoma likely to respond to ICI. Ongoing clinical trials are incorporating TLSs as a selection criterion for ICI therapy, with results anticipated soon. While TLS identification still requires tumor biopsies, the advantage of LIPI, being a non-invasive tool, holds promise for long-term monitoring of patients with cancer to detect disease progression or recurrence. Combining the low-cost, blood-based LIPI with tissue biomarkers could enhance predictive accuracy. Further research is needed on the association between TLS and other blood-based biomarkers such as LIPI.

Given that LIPI is based on the dNLR, its relevance can be biologically explained by the established clinical significance of elevated neutrophils in tumor progression. Neutrophils can facilitate tumor growth by remodeling the extracellular matrix, promoting angiogenesis, and releasing growth factors and specific enzymes that aid in tumor invasion and metastasis [27,28,29,30]. A retrospective study on systemic inflammation in STS confirmed that an elevated neutrophil-to-lymphocyte ratio (NLR) correlates with higher tumor grade, size, and depth and the presence of distant metastasis. The same study showed that NLR levels increased in patients who experienced metastatic relapse compared with their initial diagnosis of localized disease, suggesting that NLR is a dynamic marker of systemic inflammation that evolves with disease progression in STS [31]. Neutrophils are also believed to be implicated in immunotherapy resistance, as they can suppress anti-tumor T cells, which are crucial for immunotherapy due to their role in anti-tumor activity [32,33,34]. ICB therapy aims to promote the activation of these T cells, the same cells suppressed by neutrophils [35]. Therefore, high neutrophil levels, which correlate with a poorer LIPI score, are likely associated with immunotherapy resistance. This relationship could explain the statistical impact of poor LIPI in the immunotherapy group and its association with poorer patient outcomes. While the patients in this study had a washout period before measurement of dNLR, it is important to note that in clinical practice, when measuring dNLR for screening of patients, a washout period from GCSF should be taken into account.

This study has several limitations. First, as a retrospective study, it carries inherent biases, although key endpoints such as survival, response, and toxicity were carefully documented prospectively in each trial. Second, the considerable heterogeneity in the sarcoma types and treatment methods included in the study may introduce variability in the outcomes. Third, while the findings offer valuable insights, prospective validation is necessary to confirm their applicability and generalizability. Lastly, the number of patients included may have limited the study’s power; the trends observed in our control cohort might have reached statistical significance with a larger sample size.

## 5. Conclusions

In conclusion, growing evidence suggests that neutrophils contribute to ICB resistance. Elevated neutrophil levels, reflected in a high dNLR and poor LIPI score, may indicate an immunosuppressive tumor environment prone to immunotherapy resistance. This could explain LIPI’s prognostic significance in the immunotherapy setting.

Our analysis suggests that the potential implementation of LIPI score into the clinical management of patients with STS could allow clinicians to gain valuable insights into individualized patient prognosis and subsequently tailor treatments more effectively.

## Figures and Tables

**Table 1:** Baseline characteristics of patients according to LIPI group.

	Immunotherapy-Treated Patients					Control Patients				
Variable	Whole Cohort * (N = 82)	LIPI Good (N = 30)	LIPI Intermediate (N = 26)	LIPI Poor (N = 13)	*p*	Whole Cohort (N = 126)	LIPI Good (N = 73)	LIPI Intermediate (N = 33)	LIPI Poor (N = 13)	*p*
**Age > 65**	18 (22%)	6 (20%)	8 (31%)	1 (8%)	0.26	28 (22%)	17 (23%)	7 (21%)	1 (8%)	0.54
Unknown	0	0	0	0		1	0	0	0	
**Sex**					0.43					**0.03**
Female	38 (46%)	11 (37%)	14 (54%)	6 (46%)		73 (58%)	37 (51%)	23 (70%)	11 (85%)	
Male	44 (54%)	19 (63%)	12 (46%)	7 (54%)		53 (42%)	36 (49%)	10 (30%)	2 (15%)	
**FNCLCC grade** median [IQR]	2 [2;3]	2 [1.75;3]	3 [2;3]	2 [2;3]	0.10	2 [1;3]	2 [1;3]	2 [1;3]	2 [2;3]	0.11
Unknown	22	10	5	3		28	20	4	4	
**Genomic profile**					0.08					**0.01**
Complex karyotype	44 (54%)	15 (50%)	19 (73%)	5 (38%)		62 (49%)	29 (40%)	21 (64%)	10 (77%)	
Translocation- related sarcoma	37 (46%)	15 (50%)	7 (27%)	8 (62%)		64 (51%)	44 (60%)	12 (36%)	3 (23%)	
Unknown	1	0	0	0		0	0	0	0	
**Histology**					**0.02**					0.06
Angiosarcoma	6 (7%)	2 (7%)	1 (4%)	1 (8%)		6 (5%)	4 (5%)	1 (3%)	0 (0%)	
Leiomyosarcoma	22 (27%)	6 (20%)	13 (50%)	2 (15%)		32 (25%)	15 (21%)	12 (36%)	5 (38%)	
Myxoid liposarcoma	5 (6%)	1 (3%)	1 (4%)	2 (15%)		6 (5%)	4 (5%)	2 (6%)	0 (0%)	
Synovial sarcoma	7 (9%)	1 (3%)	2 (8%)	4 (31%)		5 (4%)	3 (4%)	0 (0%)	2 (15%)	
Undifferentiated pleomorphic sarcoma	5 (6%)	2 (7%)	3 (12%)	0 (0%)		11 (9%)	6 (8%)	4 (12%)	1 (8%)	
Well/de-differentiated liposarcoma	9 (11%)	7 (23%)	2 (8%)	0 (0%)		45 (36%)	29 (40%)	11 (33%)	0 (0%)	
Other	28 (34%)	11 (37%)	4 (15%)	4 (31%)		21 (17%)	12 (16%)	3 (9%)	5 (38%)	
**Stage at inclusion**					0.39					0.66
Locally advanced	10 (12%)	2 (7%)	5 (19%)	1 (8%)		17 (13%)	9 (12%)	6 (18%)	1 (8%)	
Metastatic	72 (88%)	28 (93%)	21 (81%)	12 (92%)		109 (87%)	64 (88%)	27 (82%)	12 (92%)	
**N prior systemic lines** median [IQR]	3 [2;4]	4 [2;4]	3 [2;4]	4 [3;5]	0.19	2 [1;3]	2 [1;3]	2 [1;3]	2 [2;3]	0.79
**Prior** **anthracycline**	74 (90%)	27 (90%)	22 (85%)	13 (100%)	0.44	94 (75%)	55 (75%)	25 (76%)	11 (85%)	0.898
**Performance** **status > 0**	52 (63%)	17 (57%)	15 (58%)	13 (100%)	**0.01**	75 (60%)	33 (45%)	26 (79%)	11 (84%)	**0.001**
**dNLR > 3**	31 (38%)	0 (0%)	14 (54%)	13 (100%)	**<0.001**	27 (21%)	0 (0%)	14 (42%)	13 (100%)	**<0.001**
**LDH > N**	25 (36%)	0 (0%)	12 (46%)	13 (100%)	**<0.001**	32 (27%)	0 (0%)	19 (58%)	13 (100%)	**<0.001**
Unknown	13	0	0	0		7	0	0	0	
**Albumin > 35 g/L**	67 (78%)	26 (87%)	22 (85%)	7 (54%)	**0.05**	81 (64%)	48 (66%)	23 (70%)	7 (54%)	0.59

* Whole cohort including patients with non-evaluable LIPI score. Abbreviations: dNLR, derived neutrophil-to-lymphocyte ratio; FNCLCC, Federation Nationale des Centres de Lutte Contre le Cancer grading system; IQR, Interquartile Range; LIPI, Lung immune prognostic index.

**Table 2:** Multivariate Cox analysis for PFS.

Variable	Immunotherapy-Treated Patients			Control Patients		
	HR	95%CI	*p*	HR	95%CI	*p*
N prior systemic treatment lines > 2	1.77	(0.91–3.41)	0.09	0.82	(0.47–1.45)	0.50
Age > 65	1.56	(0.78–3.11)	0.21	0.88	(0.50–1.56)	0.66
Performance status > 0	1.04	(0.57–1.92)	0.89	1.48	(0.91–2.42)	0.12
LIPI			**0.01**			0.70
LIPI intermediate	2.21	(1.16–4.23)		0.97	(0.59–1.60)	
LIPI poor	3.89	(1.49–10.13)		1.34	(0.64–2.79)	
Albumin > 35 g/L	0.63	(0.31–1.29)	0.20	0.77	(0.46–1.29)	0.31
Myxoid liposarcoma	3.08	(0.85–11.13)	0.09	-	-	-
Other sarcoma histotypes	1.02	(0.52–2.02)	0.95	-	-	-
Synovial sarcoma	2.53	(0.88–7.27)	0.09	-	-	-
Genomic profile translocation related	-	-	-	0.59	(0.36–0.95)	**0.03**
Prior anthracyclines	-	-	-	1.76	(0.98–3.17)	0.06
Liver metastasis	-	-	-	0.88	(0.49–1.57)	0.66
Lung metastasis	-	-	-	1.46	(0.84–2.54)	0.18
Combination treatments	-	-	-	0.59	(0.36–0.95)	**0.03**
Angiosarcoma	-	-	-	1.66	(0.55–5.04)	0.37
Well/de-differentiated liposarcoma	-	-	-	1.07	(0.53–2.15)	0.85

Abbreviations: 95%CI, 95% Confidence Interval; HR, hazard ratio; LIPI, lung immune prognostic index.

## Data Availability

Data will not be made publicly available. De-identified data may be shared upon reasonable academic request to the corresponding author and will be subject to legal data transfer agreements.

## Supplementary Materials

The following supporting information can be downloaded at: https://www.mdpi.com/article/10.3390/cancers16234053/s1, Table S1: LIPI Score definition; Table S2: Comparison of LIPI in control group vs. immunotherapy group; Table S3: Univariate Cox analyses for progression-free survival; Table S4: Cox univariate OS analyses of immunotherapy cohort; Table S5: Cox univariate OS analyses of control cohort; Table S6: Multivariate Cox model for OS of immunotherapy cohort; Table S7: Multivariate Cox model for OS of control cohort; Table S8: Multivariate Cox Model for OS of Immunotherapy Cohort; Table S9: Multivariate Cox Model for OS of Control Cohort; Figure S1: Population flow chart; Figure S2: Histotypes according to LIPI group; Figure S3: PFS by dNLR in immunotherapy group/PFS by dNLR in control group; Figure S4: OS by dNLR in immunotherapy group/OS by dNLR in control group.
