# Supplementary material for: Association of Lung Immune Prognostic Index (LIPI) with Disease Control Rate and Progression-Free Survival in Patients with Soft-Tissue Sarcoma Treated with Immunotherapy in Early-Phase Trials
Source: Cancers (Basel). 2024 Dec 3;16(23):4053. doi: 10.3390/cancers16234053 (PMC11640131; doi:10.3390/cancers16234053)
Supplement: Supplementary file 1 [file cancers-16-04053-s001.zip › cancers-3300638-supplementary.pdf]

Supplemental Material

Table S1. LIPI Score Definition.

LIPI	
**Good**	dNLR <3 and LDH<Normal
Intermediate	dNLR >3 **or** LDH>Normal
Poor	dNLR >3 and LDH>Normal

dNLR, derived neutrophil-to-lymphocyte ratio = derived neutrophils/ (leukocytes minus neutrophils); LIPI, Lung Immune Prognostic Index; LDH, lactate dehydrogenase level.

Table S2. Comparison of LIPI in control group vs immunotherapy group.

Covariate	Control patients (N=126)	Immunotherapy-treated patients (N=82)
**Center**		
Centre Leon Berard	50 (40%)	29 (35%)
Gustave Roussy	76 (60%)	53 (65%)
**Sex**		
Female	73 (58%)	38 (46%)
Male	53 (42%)	44 (54%)
**Grade FNLCC**		
Median [IQR]	2 [1;3]	2 [2;3]
NA	28	22
**Genetic profile**		
Complex karyotype	62 (49%)	44 (54%)
Translocation related sarcoma	64 (51%)	37 (46%)
**Stage inclusion**		
Locally advanced	17 (13%)	10 (12%)
Metastatic	109 (87%)	72 (88%)
**Number of prior lines**		
Median [IQR]	2 [1;3]	3 [2;4]
**Liver Metastasis**	26 (21%)	21 (26%)
**Lung Metastasis**	56 (44%)	65 (79%)
**Prior Anthracylines**		
No	32 (25%)	8 (10%)
Yes	94 (75%)	74 (90%)
**Combination treatment** *		
No	88 (70%)	11 (13%)
Yes	38 (30%)	71 (87%)
**Age at inclusion**		
Median [IQR]	57.5 [50;65]	55 [45.25;62.75]
**Performance status category**		
0	50 (40%)	30 (37%)
1 or more	75 (60%)	52 (63%)
NA	1	
**LIPI**		
Good	73 (61%)	30 (43%)
Intermediate	33 (28%)	26 (38%)
Poor	13 (11%)	13 (19%)
NA	7	13

dNLR, derived neutrophil-to-lymphocyte ratio; FNCLCC, Federation Nationale des Centres de Lutte Contre le Cancer grading system; ICB, Immune Checkpoint Inhibitor; LIPI, Lung Immune Prognostic Index; RMH, Royal Marsden Hospital; UPS, Undifferentiated Pleomorphic Sarcoma;

WD/DD, Well-differentiated/ Dedifferentiated.
*Combination treatment: More than one molecule.

Table S3. Best response by LIPI group with Stable Disease > 8 weeks.

	Whole	Good	Intermediate	Poor	p
**Immunotherapy**					**<0.001**
DCR*	39 (48%)	22 (73%)	11 (42%)	0 (0%)	
NA	2			1	
**Control**					0.93
DCR	78 (62%)	48 (66%)	21 (64%)	6 (46%)	
NA	10	4	3	3	

*Disease Control Rate = stable disease + partial response + complete response.

DCR, Disease Control Rate; NA, Not Available; LIPI, Lung Immune Prognostic Index.

Table S4. Univariate Cox Analyses for Progression-Free Survival.

Variable	Immunotherapy			Control		
	HR	95%CI	p	HR	95%CI	p
Centre Gustave Roussy	1.54	1-2.58	0.11	1.57	1.07-2.31	0.02
Sex Male	1.05	1-1.7	0.85	0.91	0.62-1.32	0.62
FNLCC Grade	1.11	1-1.7	0.61	1.40	1.09-1.79	0.01
Genetic profile Translocation related sarcoma	0.87	1-1.4	0.57	0.60	0.41-0.88	0.01
Metastatic at inclusion	1.42	1-3.311	0.42	1.57	0.89-2.76	0.12
N prior lines >2	2.01	1.2-3.36	0.01	1.44	0.97-2.15	0.07
Prior anthracycline	3.47	1.3-9.626	0.02	2.15	1.36-3.41	0.001
N metastasis >2	1.16	1-1.9	0.54	1.34	0.86-2.08	0.20
Liver metastasis	1.48	0.88-2.50	0.14	1.45	0.93-2.28	0.10
Lung metastasis	1.32	1-2.44	0.37	1.71	1.16-2.51	0.006
Combination treatment*	1.93	1-4.292	0.11	0.56	0.37-0.84	0.006
Age >65	1.47	1-2.52	0.16	0.62	0.39-0.99	0.04
Performance Status > 0	2.15	1.3-3.61	0.004	1.58	1.07-2.34	0.02
dNLR >3	2.61	1.6-4.319	<0.001	1.25	0.80-1.98	0.33
LDH Low	0.38	0-1	<0.001	0.64	0.42-0.98	0.04
LIPI intermediate	2.21	1.2-3.962	0.01	1.09	0.70-1.71	0.70
LIPI poor	7.09	3.302-15.229	<0.001	2.17	1.18-3.97	0.01
Albumin >35 g/L	0.37	0-1	0.001	0.622	0.42-0.92	0.02
RMH Score	1.93	1.4-2.6	<0.001	1.59	1.27-1.98	<0.001
Toxicity Grade 3-5	1.61	1-2.65	0.06	0.7	0.48-1.02	0.06

95%CI, 95% Confidence Interval; dNLR, derived neutrophil-to-lymphocyte ratio; FNCLCC, Federation Nationale des Centres de Lutte Contre le Cancer grading system; HR, Hazard Ratio; LDH, lactate dehydrogenase; LIPI, Lung Immune Prognostic Index; RMH, Royal Marsden Hospital;WBC,

White blood cells.
* Combination treatment: More than one molecule.

Table S5. Overall Survival and Progression-Free Survival of Patients with Stable Disease > 8 Weeks in Immunotherapy and Control Cohorts.

	N patients	N events	Median, months (95%CI)
	**Immunotherapy**	**(n=82)**	
**Overall survival**			
SD disease	37	15	16.7 (11.2-NR)
SD and LIPI Good	20	5	NR (16.7-NR)
SD and LIPI Intermediate	12	8	10.7 (5.6-NR)
SD and LIPI Poor	1	1	3.6 (NR-NR)
**Progression free survival**			
SD disease	37	29	4.9 (4.0-6.2)
SD and LIPI Good	20	16	5.0 (4.0-NR)
SD and LIPI Intermediate	12	11	4.3 (2.7-NR)
SD and LIPI Poor	1	1	1.6 (NR-NR)
	**Control**	**(n=126)**	
**Overall survival**			
SD disease	66	46	21.8 (16.0-31.9)
SD and LIPI Good	40	25	30.1 (17.3- 61.0)
SD and LIPI Intermediate	17	12	14.5 (10.3-NR)
SD and LIPI Poor	6	6	10.4 (4.3-NR)
**Progression free survival**			
SD disease	66	57	5.6 (4.4-7.6)
SD and LIPI Good	40	34	5.6 (4.1-8.2)
SD and LIPI Intermediate	17	14	5.4 (3.5-NR)
SD and LIPI Poor	6	6	3.4 (3.0-NR)

95%CI, 95% Confidence Interval; LIPI, Lung Immune Prognostic Index; NR, Not Reached; SD, Stable Disease

Table S6. Cox Univariate OS Analyses of Immunotherapy Cohort.

	HR	95%CI	p
Centre Gustave Roussy	1.11	0.59 -2.08	0.75
Sex Male	1.16	0.64 -2.11	0.63
FNLCC Grade	1.47	0.88 -2.46	0.15

Genetic profile<br>Translocation related sarcoma	1.19	0.64 -2.20	0.59
Metastatic stage at inclusion	3.12	0.74 -13.17	0.12
N prior lines >2	1.73	0.91 -3.31	0.10
Prior anthracycline	3.02	0.92 -9.93	0.07
N metastasis >2	1.29	0.7 -2.36	0.42
Lung metastasis	2.31	0.96 -5.55	0.06
Combination treatment*	2.10	0.81 -5.48	0.13
Age >65	1.42	0.71 -2.82	0.33
Performance status > 0	2.63	1.32 -5.24	**0.01**
dNLR >3	4.31	2.31 -8.03	**<0.001**
LDH low	0.21	0.10 -0.41	**<0.001**
LIPI intermediate	3.49	1.51 -8.10	**0.004**
LIPI poor	28.47	10.15 -79.81	**<0.001**
Albumin >35 g/L	0.16	0.076 -0.33	**<0.001**
RMH	3.37	2.22 -5.11	**<0.001**
Toxicity grade 3-5	2.92	1.54 -5.54	**0.001**
Angiosarcoma	1.61	0.49 -5.32	0.43
Leiomyosarcoma	1.02	0.52 -1.99	0.96
Myxoid liposarcoma	2.79	0.98 -7.90	0.05
Other	0.45	0.22 -0.93	**0.03**
Synovial sarcoma	5.79	2.36 -14.21	**<0.0001**
UPS	0.28	0.04 -2.03	0.21
WD/DD liposarcoma	1.22	0.51 - 2.91	0.65

95%CI, 95% Confidence Interval; dNLR, derived neutrophil-to-lymphocyte ratio; FNCLCC, Federation Nationale des Centres de Lutte Contre le Cancer grading system; HR, Hazard Ratio; LDH, lactate dehydrogenase; LIPI, Lung Immune Prognostic Index; RMH, Royal Marsden Hospital; UPS, Undifferentiated Pleomorphic Sarcoma; WD/DD, Well-differentiated/ Dedifferentiated.
* Combination treatment: More than one molecule.

Table S7. Cox Univariate OS Analyses of Control Cohort.

	**HR**	**95%CI**	**p**
Centre Gustave Roussy	0.985	0.664-1.461	0.939
Sex Male	1.016	0.686-1.504	0.937
FNLCC Grade	1.429	1.125-1.816	**0.003**
Genetic profile<br>Translocation related sarcoma	0.704	0.472-1.051	0.086
Metastatic at inclusion	1.731	0.945-3.173	0.076
N prior lines >2	1.165	0.77-1.764	0.469
Prior anthracycline	1.982	1.231-3.192	**0.005**
N metastasis >2	1.358	0.815-2.263	0.24
Lung metastasis	1.637	1.097-2.441	**0.016**
Combination treatment*	0.826	0.54-1.264	0.379
Age >65	0.763	0.47-1.237	0.272
Performance status > 0	2.197	1.443-3.347	**<0.001**
dNLR >3	1.394	0.874-2.223	0.164
LDH low	0.42	0.271-0.652	**<0.001**
LIPI intermediate	1.226	0.771-1.949	0.39
LIPI poor	4.022	2.197-7.362	**<0.001**

Albumin >35 g/L	0.472	0.315-0.708	**<0.001**
RMH	2.077	1.611-2.677	**<0.001**
Toxicity grade 3-5	1.504	1.016-2.228	**0.041**
Angiosarcoma	1.12	0.41-3.062	0.825
Leiomyosarcoma	1.257	0.756-2.089	0.378
Myxoid liposarcoma	0.642	0.235-1.755	0.388
Other	0.84	0.475-1.488	0.551
Synovial sarcoma	2.327	0.937-5.779	0.069
UPS	1.416	0.702-2.854	0.331
WD/DD liposarcoma	0.811	0.53-1.242	0.336

95%CI, 95% Confidence Interval; dNLR, derived neutrophil-to-lymphocyte ratio; FNCLCC, Federation Nationale des Centres de Lutte Contre le Cancer grading system; HR, Hazard Ratio; LDH, lactate dehydrogenase; LIPI, Lung Immune Prognostic Index; RMH, Royal Marsden Hospital; UPS, Undifferentiated Pleomorphic Sarcoma; WD/DD, Well-differentiated/ Dedifferentiated.
* Combination treatment: More than one molecule.

Table S8. Multivariate Cox Model for OS of Immunotherapy Cohort.

	**HR**	**95%CI**	**p**
N lines > 2	1.29	0.54-3.06	0.56
Synovial sarcoma	3.78	1.29 -11.08	**0.015**
Liver metastasis	1.41	0.66-3.02	0.37
Performance status ≥1	1.47	0.61 -3.58	0.39
Albumin > 35 g/L	0.23	0.1 -0.54	**0.001**
LIPI intermediate	2.94	1.15 -7.49	**<0.0001**
LIPI Poor	15.68	4.89 -50.21	**<0.0001**

95%CI, 95% Confidence Interval; HR, Hazard Ratio; LIPI, Lung Immune Prognostic Index.

Table S9. Multivariate Cox Model for OS of Control Cohort.

	**HR**	**95%CI**	**p**
Translocation related sarcoma	0.72	0.40 -1.29	0.27
Age > 65	1.22	0.66 -2.24	0.53
Prior anthracyclines	1.32	0.7 -2.50	0.39
N prior lines > 2	0.76	0.42-1.38	0.375
Synovial sarcoma	3.12	1.07 -9.09	**0.038**
Liver metastasis	0.99	0.50-1.99	0.99

Lung metastasis	1.47	0.84-2.58	0.18
Performance status ≥1	1.92	1.15-3.22	**0.01**
Albumin > 35 g/L	0.44	0.25-0.75	**0.002**
LIPI intermediate	1.06	0.61-1.84	0.07
LIPI poor	2.34	1.13-4.86	

95%CI, 95% Confidence Interval; HR, Hazard Ratio; LIPI, Lung Immune Prognostic Index.

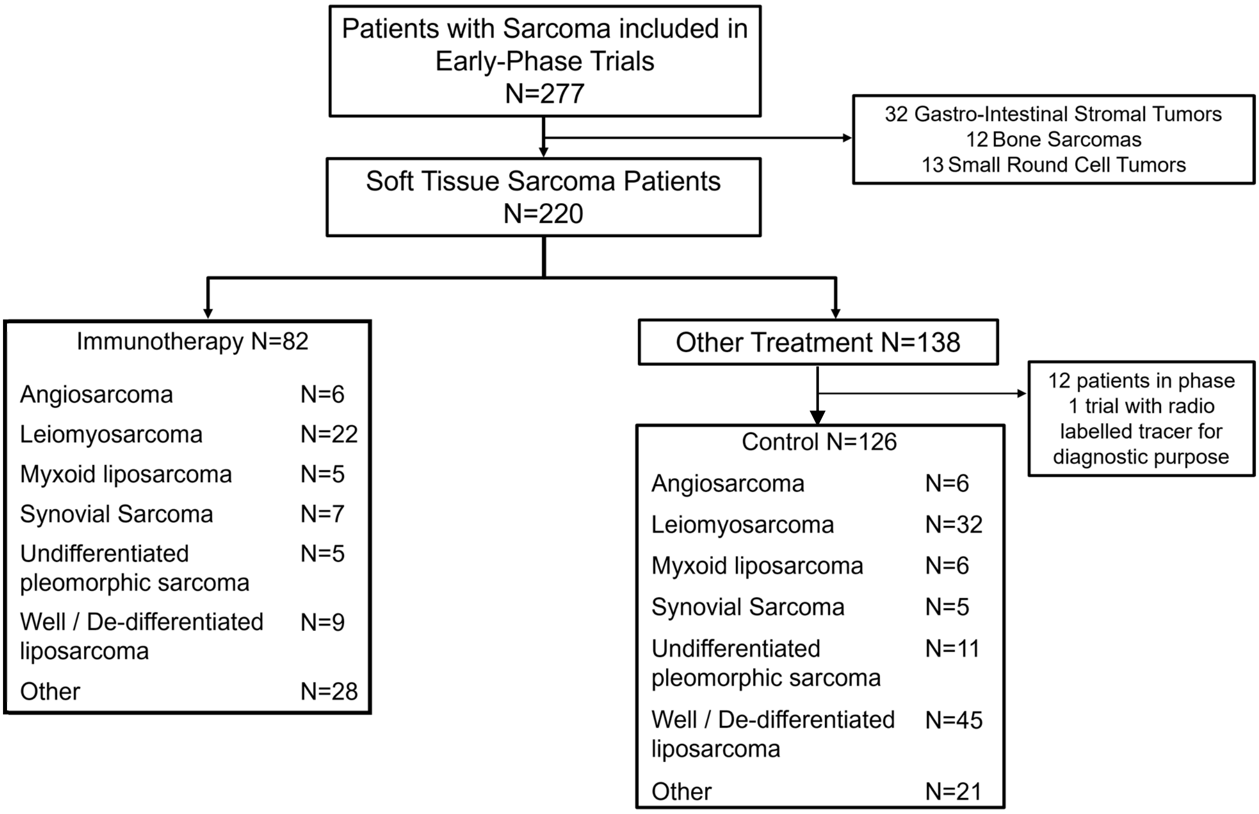

Figure S1. Population flowchart.

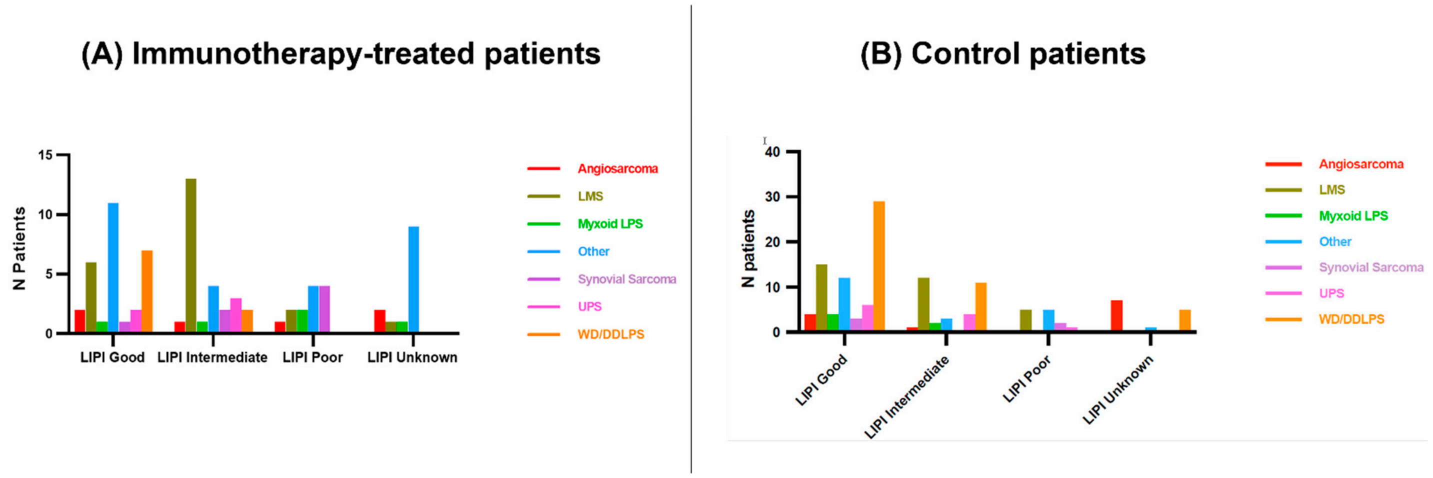

Figure S2. Histotypes according to LIPI group; LMS, Leiomyosarcoma; LPS, liposarcoma; UPS,Undifferentiated pleomorphic sarcoma; WD/DD LPS,Well / De-differentiated liposarcoma.

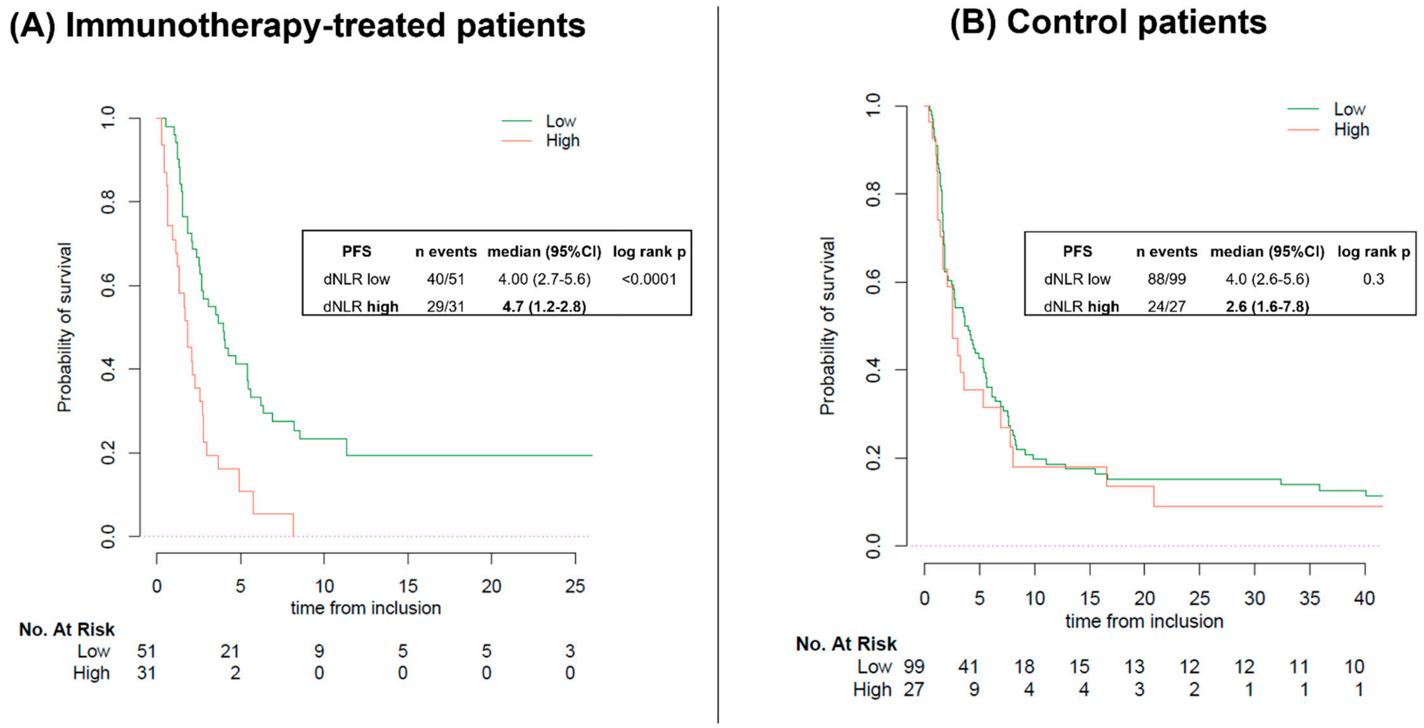

Figure S3. PFS by dNLR in immunotherapy group/ PFS by dNLR in control group. 95%CI, 95% Confidence Interval; PFS, Progression-Free Survival.

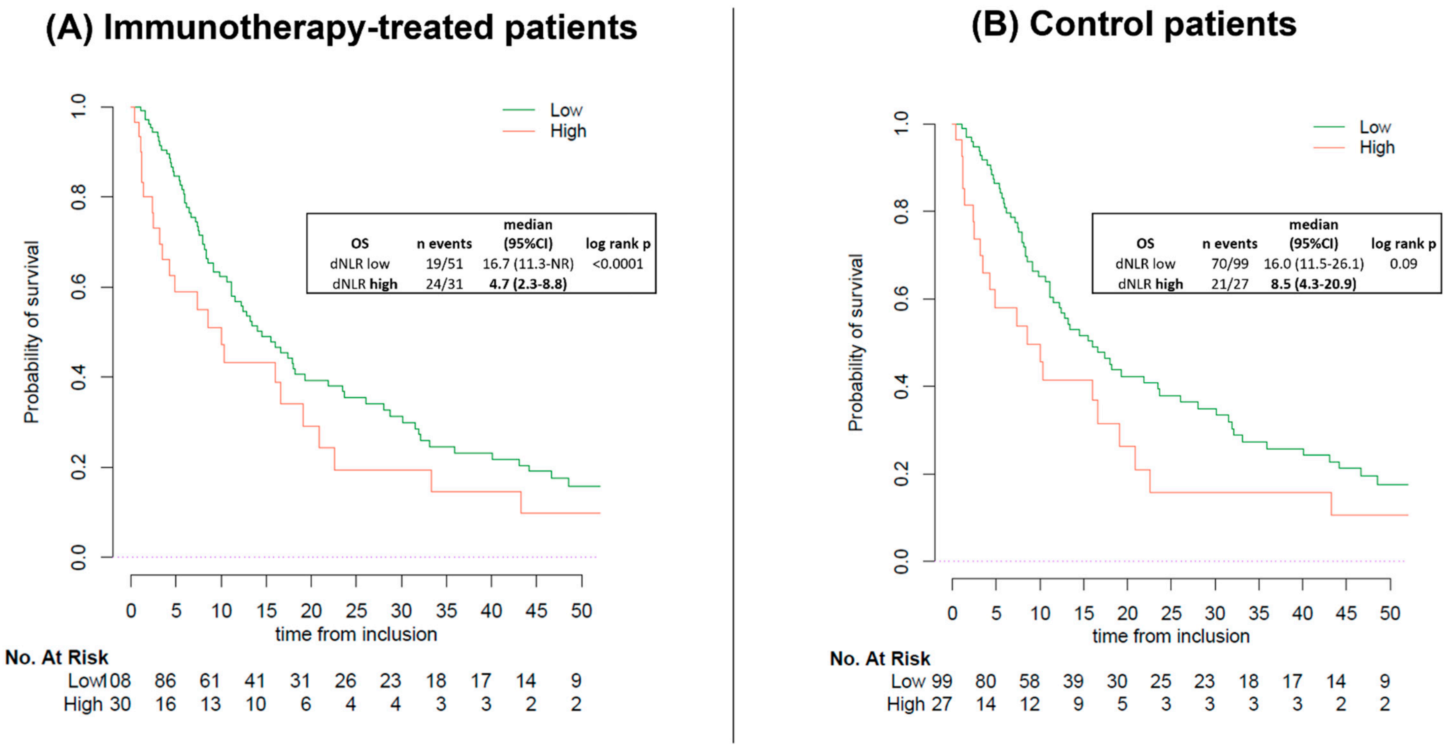

Figure S4. OS by dNLR in immunotherapy group/ OS by dNLR in control group. 95%CI, 95% Confidence Interval; OS, Overall Survival.
